# Simulations of Myenteric Neuron Dynamics in Response to Mechanical Stretch

**DOI:** 10.1155/2020/8834651

**Published:** 2020-10-13

**Authors:** Donghua Liao, Jingbo Zhao, Hans Gregersen

**Affiliations:** ^1^Mech-Sense, Department of Gastroenterology, Aalborg University Hospital, Aalborg, Denmark; ^2^Giome Academia, Department of Clinical Medicine, Aarhus University, Aarhus, Denmark; ^3^Standard (Chongqing) Pathological Diagnosis Center, No. 8 Xiyuan North Road Shapingba District, Chongqing, China; ^4^Calmi2, San Diego, CA, USA; ^5^GIOME, Discovery Bay, Hong Kong

## Abstract

**Background:**

Intestinal sensitivity to mechanical stimuli has been studied intensively in visceral pain studies. The ability to sense different stimuli in the gut and translate these to physiological outcomes relies on the mechanosensory and transductive capacity of intrinsic intestinal nerves. However, the nature of the mechanosensitive channels and principal mechanical stimulus for mechanosensitive receptors are unknown. To be able to characterize intestinal mechanoelectrical transduction, that is, the molecular basis of mechanosensation, comprehensive mathematical models to predict responses of the sensory neurons to controlled mechanical stimuli are needed. This study aims to develop a biophysically based mathematical model of the myenteric neuron with the parameters constrained by learning from existing experimental data. *Findings*. The conductance-based single-compartment model was selected. The parameters in the model were optimized by using a combination of hand tuning and automated estimation. Using the optimized parameters, the model successfully predicted the electrophysiological features of the myenteric neurons with and without mechanical stimulation.

**Conclusions:**

The model provides a method to predict features and levels of detail of the underlying physiological system in generating myenteric neuron responses. The model could be used as building blocks in future large-scale network simulations of intrinsic primary afferent neurons and their network.

## 1. Introduction

Intrinsic primary afferent neurons (IPANs) in the myenteric plexus comprise an important group of mechanosensory neurons for primary neural control of gastrointestinal motility [1]. It is a ganglionated network within the myenteric plexus and with projections into the mucosa [2–4]. The sensory neurons respond to changes in the chemical content of the intestinal lumen and wall stretch or tension [5, 6]. However, the ability to sense different stimuli in the gut and translate those to physiological outcomes relies on the mechanosensory and transductive capacity of the intrinsic intestinal nerves.

IPANs exhibit the Dogiel type II morphology and intracellular recordings have shown that they possess electrophysiological properties of after-hyperpolarization (AHP) [2, 7]. The dynamic response properties of the IPANs from afferent nerve bundles to single neurons have been extensively studied [2, 8, 9]. Both single neurons and afferent nerve bundles exhibited similar responses in thresholds for activation and discharge rate to standard test protocols such as ramp or step distension [9–12]. However, many questions regarding functional aspects of the mechanical stimulus-to-mechanosensitive channels transfer characteristics of these receptors remain unknown. Mathematical modeling provides a unique framework that allows one to link a highly complex relation between natural sensory stimuli and neuronal responses. Consequently, comprehensive mathematical models are needed for better understanding of the stimulus-response interaction in myenteric neurons.

Numerous attempts have been made to mathematically describe the functional input–output properties of intrinsic sensory neurons of the myenteric plexus related to motility and intestinal reflexes [13–18]. Those models vary considerably in complexity and their ability to adequately address the intricacies of the structural and ionic mechanisms associated with the mechanical stimulus. Those efforts have concentrated on modeling IPAN function and networks by constructing biophysically realistic compartment models of the individual neurons in the circuit. Parameters that relate to the underlying biophysics of the real neuron are essential to the model. However, not all the parameters can be measured directly from experiments. Therefore, some parameters must be tuned to match the experimentally observed input–output relation of the neuron by solving nonlinear optimization problems.

In this study, we developed a biophysically based mathematical model of the Dogiel type II myenteric neuron with the mechanosensitive channel taking into account [16, 19, 20]. Parameters related to biophysical properties of the slow after-hyperpolarization (sAHP) and medium after-hyperpolarization (mAHP) neurons in the myenteric plexus of the pig small intestine [21] were optimized by fitting measured electrophysiological features to the model calculation. The sAHP and mAHP are two electrophysiological subpopulations in the porcine Dogiel type II neurons, where the sAHP neuron needs longer time to reach maximum amplitude and with longer duration of the AHPs [21]. Furthermore, the properties of the mechanosensitive channel were investigated by optimizing the model to spike discharges of a single neuron in an elongated tissue strip [2]. The developed model can potentially be used as building blocks in future large-scale network simulations of IPANs.

## 2. Material and Methods

In this study, a mathematical model of the Dogiel type II myenteric neuron was developed based on recently published models from Osorio et al. and Korogod et al. [16, 19]. The model was used to estimate the biophysical properties of myenteric plexus neurons exposed to mechanical stretch. Estimations were conducted by optimizing the model to previously recorded electrophysiological features of the myenteric neurons in studies done by Kunze et al. and Cornelissen et al. [2, 21].

### 2.1. Myenteric Neuron Model

The computational myenteric neuron model is a single-compartment model, containing the channels conducting passive leakage current *I*_leak_, mechanosensory current *I*_*m*_, and seven voltage-gated currents described by Hodgkin–Huxley type equations. There were three sodium currents (TTX-sensitive current *I*_Na-TTX_, the TTX-resistant current *I*_Nav1.5_, and *I*_Nav1.9_), two potassium currents (delayed rectifier *I*_Kdr_ and M-type *I*_KM_), the N-type calcium current (*I*_CaN_), and nonspecific currents through hyperpolarization activated channels *I*_*h*._

The dynamic of the membrane potential *V* can be described by the integrated function of the nine ionic channels as

(1) $$\begin{array}{c} C.\frac{\text{d}V}{\text{d}t}=-\sum I_{i}+I_{\text{app}}, \end{array}$$

where ∑*I*_*i*_=*I*_leak_+*I*_Na−*TTX*_+*I*_Nav1.5_+*I*_Nav1.9_+*I*_Kdr_+*I*_KM_+*I*_CaN_+*I*_*h*_+*I*_*m*_, *I*_app_ is the external current and *C*=1*μ*F/cm^2^ is the membrane capacitance of the neuron. The Hodgkin–Huxley type equations for each ionic current are listed in the appendix.

### 2.2. Biophysical Properties Estimation and Optimization

The biophysical properties of the neuron were described mathematically through the model parameters. The parameters were optimized by using a combination of hand tuning and automated estimation in finding the minimum of the objective function of

(2) $$\begin{array}{c} J\left(\theta \right)=\;\sum {\left[V_{\text{exp}}\left(t_{i}\right)-V_{\text{mod}}\left(t_{i},\theta \right)\right]}^{2}, \end{array}$$

where *θ* denotes the vector of the maximum conductance in the current calculations of the nine ionic channels (equations (1) and (A.1)–(A.22)), *V*_exp_(*t*_*i*_) the membrane potential (MP) recorded at time *t*_*i*_, and *V*_mod_(*t*_*i*,*θ*_) the corresponding model calculated MP at the time.

The single-compartment model was generated by using NEURON 7.67 (https://neuron.yale.edu/neuron/). Parameter estimation was done by using the optimization algorithm principal axis method embedded in the Multiple Run Fitter in NEURON [22]. During the optimization, the maximum conductance of each ionic channel was variable whereas the kinetic parameters of each channel were fixed [16, 23]. Collectively, nine free parameters were adjusted during the optimization; that is, all free parameters were varied for matching the experimental recordings within a given tolerance.

The model was obtained by optimizing the membrane potential denoted in equation (1) to the experimental recordings of the MP in Dogiel type II neurons. Moreover, for demonstrating the reliability of the optimized parameters, the model generalizations were compared to the experimental recordings that were not included in the optimization process. The experimental recordings were recaptured from previous studies done by Cornelissen et al. [21] and Kunze et al. (the neurons in a tissue strip under 20% and 40% longitudinal stretch in [2]. The recordings were digitized from the published figures by using homemade MATLAB image processing subroutines (MATLAB R2017b, MathWorks).

### 2.3. Statistics

All the results were expressed as median and Interquartile range unless otherwise stated. Wilcoxon Signed-Rank Test method was used to compare the parameters difference between sAHP and mAHP ((parameters at mAHP – parameters at sAHP)/parameters at sAHP) and that between sAHP and the neuron under stretch ((parameters at stretched neuron – parameters at sAHP)/parameters at sAHP). The statistical analysis was done using SigmaPlot version 11.0 (Systat Software, Inc., Germany). The results were considered significant when *p* < 0.05.

## 3. Results

### 3.1. Model Estimation on the Morphological Dogiel Type II Neuron

**Figure 1:**
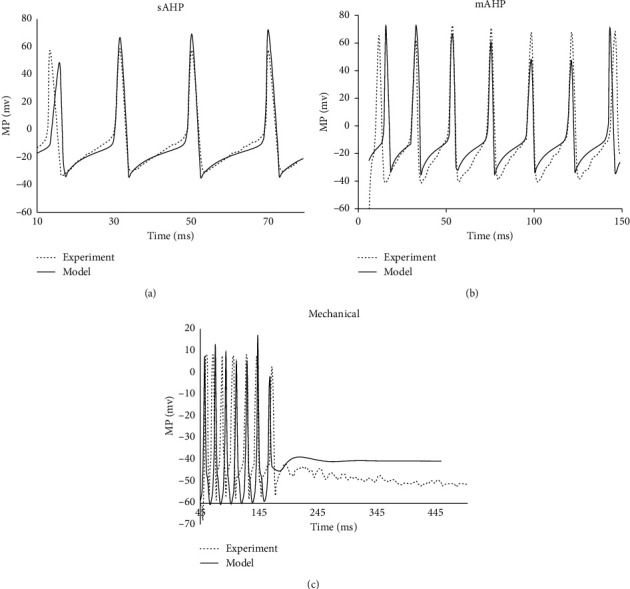
Optimizations of the model to experimentally recorded membrane potentials. (a) Comparison between the experimental recordings (dashed line) of the porcine sAHP neuron, under 100 pA, with 70 ms injection of rectangular electrical current pulses and the model response (solid lines) to the same input. The optimized conductances are listed in Table 1: sAHP neuron. (b) Comparison between the experimental recording (dashed line) of the porcine mAHP neuron, to 60 pA, with 140 ms injection of rectangular electrical current pulses and the model response (solid lines) to the same input. The optimized conductances are listed in Table 1: mAHP neuron. (c) Comparison between the experimental recording (dashed line) from a neuron in a tissue strip under 20% longitudinal stretch and with 200PA, 500 ms injection of the current pulse and the model response (solid lines) to the same input. The optimized conductances are listed in Table 1: neuron under stretch. The experimental recordings of the sAHP and mAHP were reproduced from Figure 1(b) (sAHP) and Figure 3C (mAHP) in the study by Cornelissen et al. [21], and the recordings on the mechanical stretch neuron were from Figure 5Ba in the study by Kunze et al. [2].

The electrophysiological features of sAHP and mAHP neurons with Dogiel type II morphology in the myenteric plexus of pig small intestine were selected for the model optimization (Figures 1(a) and 1(b)). The optimization processes were done by fitting the model to experimentally recorded membrane potential (MP) of the neurons with the injection of rectangular electrical current pulses. Since the external current pulse was the only stimulus on the neurons, the mechanosensitive channel current in equation (1) was not included in the optimization. Figures 1(a) and 1(b) show the model simulated MP from both sAHP and mAHP neurons closely reproduced the recorded MPs. The determined parameters are listed in Table 1.

### 3.2. Model Estimation of the Neuron During Mechanical Stretch

The MPs from Dogiel type II myenteric neurons of the guinea pig ileum were selected to estimate the model with the mechanosensitive channel current. The MPs were recorded from neurons in a tissue strip under 20% longitudinal stretch and with an injection of 500 ms current pulse. The fitted MP curves matched well with the experimental recordings (Figure 1(c)). The optimized biophysical parameters for the model are listed in Table 1, where the mechanosensitive channel was included in the model and the maximum conductance of the channel was obtained.

In Table 1, the parameters difference between sAHP and mAHP neurons was significantly smaller than that between sAHP neuron and the stretched neuron (0.094 (0.07, 0.17) vs. 2.71 (0.78, 10.6), *p* < 0.01), indicating species dependency of the parameters in the neuron model.

### 3.3. Model Evaluation

**Figure 2:**
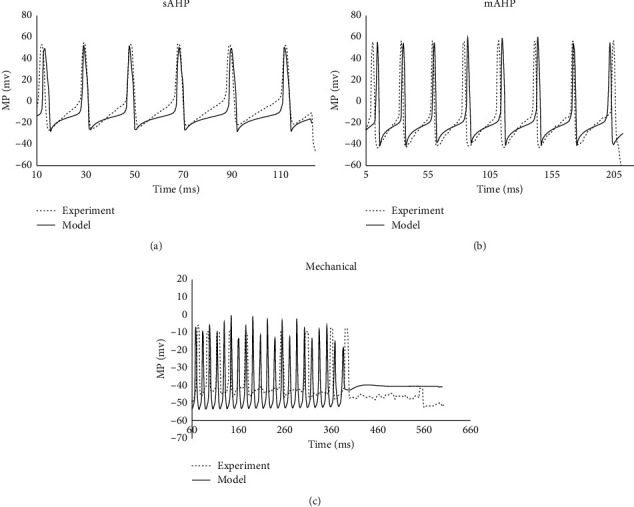
Model generalization: to test the optimized model, we compared the model responses (solid lines) to a new input current and stretch ratio with the corresponding experimentally recorded membrane potentials of three different neurons (dashed lines). These experimental traces have not been used during the optimization. (a) Comparison between the experimental recordings (dashed line) of the porcine sAHP neuron, to 60 pA, with 120 ms injection of rectangular electrical current pulses and the model prediction (solid lines) to the same input by using the parameters listed in Table 1: sAHP neuron. (b) Comparison between the experimental recordings (dashed line) of the porcine mAHP neuron, to 50 pA, with 200 ms injection of rectangular electrical current pulses and the model prediction (solid lines) to the same input by using the parameters listed in Table 1: mAHP neuron. (c) Comparison between the experimental recordings (dashed line) from a single Dogiel type II neuron in the tissue strip under 40% longitudinal stretch and 100PA with 500 ms injection of the current pulse and the model response (solid lines) to the same input by using the parameters listed in Table 1: neuron under stretch, where *G*_m_ = 0.0838 S/cm^2^ was used for the model generalization. The experimental recordings of sAHP and mAHP were reproduced from Figure 1C (sAHP) and Figure 3B (mAHP) in the study by Cornelissen et al. [21]. The mechanical stretch neurons were from Figure 5Bb in the study by Kunze et al. [2].

The obtained models were tested by the generalization of the models to input currents and the longitudinal stretch ratio that were not included in the optimization process (Figure 2). As the maximum conductance of the mechanosensitive channel relates to the longitudinal stretch ratio (equations (A.9) and (A.10)), a new maximum conductance of the mechanosensitive channel was selected for generalization of the mechanical model. Nevertheless, the remaining parameters for the mechanical model and all parameters for sAHP and mAHP neuron models were the same as in 1. Figure 2 shows the model predicted well for sAHP, mAHP neurons (Figures 2(a) and 2(b)). For the neuron under mechanical stretch (Figure 2(c)), the model can predict some features of the recorded MP. For example, when the tissue was stretched longitudinally from 20% to 40%, the neuron responses state changed from a rapidly accommodating state (neurons in which action potential firing ceased with the first 250 ms) to the slowly accommodating state (neurons that discharged action potentials for more than 250 ms), and the discharge rate increased from 12 Hz to 42 Hz. Hence, Figure 2 shows that the developed Dogiel type II neuron model can be used to predict electrophysiological features of the neurons with and without mechanical stimulation, demonstrating the reliability of the model.

## 4. Discussion

In this study, we have developed a biophysically based mathematical model of the myenteric neuron with the mechanosensitive channel activity taken into account. The proposed framework can be used to study the biophysical properties of myenteric plexus neurons exposed to mechanical stimuli. The computational analysis showed that the conductance of the mechanosensitive channel was associated with changes in the electric state of the neuron. The model can simulate the electrophysiological features of the Dogiel type II myenteric neurons at the ionic channel level.

The enteric neurons include IPANs and a variety of interneurons and motor neurons [3, 24]. Microelectrode studies have indicated that IPANs are AHP/Dogiel type II neurons that respond to mechanical stretch or distortion [25, 26]. Studies in other sensory systems suggested the mechanoreceptors convert mechanical stimuli to receptor potentials via activation of ionic channels. The opening of the mechanosensitive channel is selective to sodium or potassium ions and the channel activation was voltage-dependent [17, 27, 28]. However, studies on the mechanosensitive channel of putative aortic baroreceptor neurons found that the channel currents appeared to be linear to the voltage and the channel opening frequency increased with pressure. Furthermore, it was unlikely that channel activity was caused by the activation of voltage-gated conductance [29]. Based on findings from the aortic baroreceptor neuron studies [29], we built the mechanosensitive channel function as that in equations (A.9) and (A.10).

Conductance-based compartment neuron models have been increasingly used in simulations of the myenteric neurons [1, 14, 16–19, 30]. Compared with previous mathematical models of the myenteric neurons, we moved a step further by including the mechanosensitive channel in the model and recreating the MPs of the single Dogiel type II sensory neuron exposed to mechanical stimulus. In the aforementioned computational frameworks, parameters for describing every ion channel that may exist in the neuron membrane were needed for the modeling analysis. For obtaining satisfactory matching between the model and actual experimental recordings, the simulation must be repeated by tuning the parameters [31, 32]. In this study, we defined an objective function at each time step for the parameters to be evaluated by running the optimization algorithm. Hence, the biophysical parameters for each ionic channel in three types of myenteric neurons can be attained by optimizing the defined model to experimental recordings from the neurons. These data allowed us to predict the electrophysiological properties of the neurons from biophysical aspects of view. In the model generation, the electrical features of neurons under mechanical stretch were simulated and compared with the experimental recordings in Kunze's study [2]. Only sparse data exist on the electrical behavior of single Dogiel type II myenteric sensory neuron under the mechanical stimulus. Furthermore, to the best of our knowledge, the Kunze study is the only study that investigated the electrical features of the same Dogiel type II myenteric neuron under different mechanical stretches. The model can be extended to fit other Dogiel type II myenteric neurons under mechanical stimulation when new experimental data are available.

For the developed neuron models in this study, the ionic channel distributions and the kinetic properties of each channel were based on previous studies on mice myenteric neurons [16, 19]. In the study done by Osorio et al., they experimentally defined the properties and distribution of TTX-R Na + currents in neurons from intact mouse myenteric ganglia. Furthermore, based on their experimental data, they built a single-compartment model of a Dogiel type II mouse myenteric neuron [19]. The combined experimental and computer simulation studies showed that tetrodotoxin-resistant sodium channels were key determinants of the electrical responsiveness of mouse myenteric neurons. Korogod et al. further developed the computational model by Osorio by providing detailed biophysical properties of the channels conducting inward Na^+^ and Ca^2+^ currents in the prototype cells and relate them to the neuron's electroresponsiveness [16]. In the current study, we developed the single-compartment model of a Dogiel type II neuron from Osorio's and Korogod's models by adding the mechanosensitive channel into the model. By using the model to fit the experimentally recorded traces from three Dogiel type II neurons, we obtained the biophysical properties of these neurons. With the proposed neuron models, we can investigate the contributions of different ionic channels to the total current and further improve the single-compartment neuron model to specific Dogiel type II myenteric neurons in future experimental studies.

In the model by Korogod, for matching the simulated electrical response to AP train response, the conductance of different channels was manually selected. Hence, the typical behavior of amplitudes of the consecutive spikes in the train cannot be reproduced. Nevertheless, in the present study, the model parameters were optimized by minimizing the least square difference between the recorded MPs and the model's response in three single Dogiel type II neurons. Good agreements between the fitted MP curves and the experimental recordings indicated the proper model parameters in all three neurons (Figure 1). Furthermore, the model generated MPs agreed well with the experimental recording, demonstrating the effectiveness of the model to the tested neurons (Figure 2).

During the optimization, we used a similar assumption to that in Korogod's study; that is, the kinetic properties of given channels remain constant whatever is their conductivity. Hence, only the maximum conductance, but not the kinetic parameters of each ionic channel, was changed and needed to be estimated optimally. However, the maximum conductance values obtained in our model were much bigger than those used in the mice model. The possible reasons for the bigger values could be as follows: (1) we simulated the model in different neurons and from different species. Comparing the parameters' difference between sAHP neuron and the neuron under stretch, the significantly smaller parameters' difference between sAHP and mAHP neurons supported the point that the parameters of the neuron model could be species-dependent, as both the sAHP and mAHP neurons were from pigs, while the neuron under stretch was from a guinea pig. However, simulations from single Dogiel type II myenteric sensory neurons in more species are needed for further verification. (2) The neuron responses are determined not only by different types of ionic channels with distinct conductance values but also by the dendritic morphology. However, by using the current single-compartment model, some morphological effects can be transferred to the conductance of various channels during the optimization. (3) It is a big challenge to constrain the density of the various membrane ion channels that play a major role in determining the electrophysiological feature of the neuron. The development of molecular biology techniques, in combination with dynamic clamp recordings, may eventually allow some of these parameters to be constrained experimentally [32]. This study is also limited by the relative lack of raw experimental data from previous studies. The experimental recordings used in this study were recaptured using an image processing algorithm from the published work [2, 21]. Due to low image resolution in the publication, it was difficult to precisely capture the points near the MP peaks. Errors in image capturing could induce some imprecision in the parameter estimation. The simulated results in Figures 1 and 2 show that the model did not fit perfectly the experimental recordings. The major reason could be that the single objective function was used for the optimization and that the comparisons between the models to the experimentally recorded MPs were done on a direct trace-to-trace basis [32]. For overcoming the shortage of the direct trace-to-trace comparisons, the multiple objective optimization method [31, 32] that allows for several objective functions corresponding to optimizations of several features of the MP responses should be considered in future modeling analysis. The model generalization to the neuron with 40% mechanical stretch showed that the neuron fired with a constant discharge rate during the firing period. However, the experimental recording showed the firing rate decreased with the stimulation. In addition to the aforementioned limitations, another reason for the different discharge feature could be caused by neuron responses recorded in a longitudinal-stretched tissue strip. The interaction between the myenteric neurons in the tissue strip may affect the recorded discharges from a single neuron. However, the model simulation showed that the neuron excitability changed from rapidly accommodating (RA) in the 20% longitudinal stretch to the slowly accommodating (SA) in the 40% longitudinal stretch; and the discharge rate increased from 12 Hz to 42 Hz. These agreed well with the experiment recordings in Kunze et al. [2] and with findings from extracellular recordings [26]. In this study, we only used one current and stretch ratio (100PA with 500 ms injection of current pulse and 40% longitudinal stretch) as input to test the optimized model. For finding the work ranges of the parameters in the models, more currents and stretch ratios are needed for testing. However, to the best of our knowledge, the data we obtained from Kunze's study are the only records of the electrical features of the same Dogiel type II myenteric neuron under a range of mechanical stretch. The model can be further tested on other Dogiel type II myenteric neurons under mechanical stimulations when new experimental data are available.

## 5. Conclusions

In conclusion, a biophysically based mathematical myenteric neuron model was built in this study. The model can simulate the electrophysiological features of the myenteric neuron and estimate the membrane ionic channel properties of the neuron. With proper experimental data, the model can be extended for parameter estimation in Dogiel type II myenteric sensory neurons from different species. Furthermore, the proposed framework can be developed to simulate the functional network, such as myenteric plexus ganglion behavior by a combination of the single neuron models with various neuronal properties and synaptic strengths [33, 34].

## Figures and Tables

**Table 1:** Estimated maximum conductance of each ionic channel for three Dogiel type II neurons.

Parameters	sAHP neuron (S/cm^2^)	mAHP neuron (S/cm^2^)	Neuron under stretch (S/cm^2^)
*G*_Na−TTX_	1.7139	1.9977	9.4565
*G*_Kdr_	9.4475	8.9442	3.668
*G*_KM_	0.081363	0.08214	0.13589
*G*_Na−19_	0.01333	0.00678	0.20347
*G*_Ca−*N*_	0.015867	0.013165	0.20074
*G*_Na−15_	0.004571	0.00416	0.048289
*G*_*L*_	0.004025	0.00363	0.0076874
*G*_*h*_	0.46796	0.5097	0.054132
**G**_**m**_			0.085052

*Note.* sAHP and mAHP neurons: slow after-hyperpolarization and medium after-hyperpolarization neurons in the myenteric plexus of pig small intestine [21]. Neuron under stretch: myenteric neurons of the guinea pig ileum under longitudinal stretch [2].

## Data Availability

The datasets supporting the conclusions of this article are included within the article and its additional files.

## Appendix

According to previous studies [16, 19], the Hodgkin–Huxley type equations for each ionic current calculation are as follows:

1. TTX-sensitive sodium current:

(A.1) $$\begin{array}{c} I_{\text{Na}-\text{TTX}}=G_{\text{Na}-\text{TTX}}.m^{3}.h.\left(V-E_{\text{Na}}\right), \end{array}$$

  where *G*_Na−TTX_ is the maximum conductance, *E*_Na_=+62mV the reversal potential for sodium current, and *V* the membrane action potential. *m* and *h* are activation and inactivation variables and satisfy the ordinary differential equations as follows:
  Activation variable *m*:

(A.2) $$\begin{array}{c} \frac{\text{d}m}{\text{d}t}=\frac{\left(m_{\infty }-m\right)}{\tau_{m}}, \end{array}$$

  where *τ*_*m*_=1/[*α*_*m*_(*V*)+*β*_*m*_(*V*)] characterizes how fast activation respond to changes in voltage, *m*_*∞*_=*α*_*m*_(*V*)/[*α*_*m*_(*V*)+*β*_*m*_(*V*)] is the activation variable at the static state. *α*_*m*_(*V*) and *β*_*m*_(*V*) are functions of the voltage and were chosen to have Boltzmann-like forms. Kinetic parameters of the function were curve-fitted from the experimental recording on single myenteric neurons done by Osorio et al. as

(A.3) $$\begin{array}{c} \alpha_{m}\left(V\right)=\frac{Q_{10}\;\cdot 0.061132\cdot \left(V+34.653\right)}{\left[1-\text{Exp}\left(-V+34.653/27.197\right)\right]},\\ \beta_{m}\left(V\right)=Q_{10}\cdot 7.3036\cdot \text{Exp}\left(-\frac{V+34.907}{6.3046}\right), \end{array}$$

  where *Q*_10_=3^(*t* − 22.0)/10.0^ and *t* is temperature in Celsius during the test.
  Inactivation variable *h*:

(A.4) $$\begin{array}{c} \frac{\text{d}h}{\text{d}t}=\frac{\left(h_{\infty }-h\right)}{\tau_{h}}, \end{array}$$

  where *τ*_*h*_=1/(*α*_*h*_+*β*_*h*_), *h*_*∞*_=*α*_*h*_/(*α*_*h*_+*β*_*h*_) has the same physical meaning as *τ*_*m*_ and *m*_*∞*_ in (A.6), and *α*_*h*_ and *β*_*h*_ are functions of the voltage, denoted as

(A.5) $$\begin{array}{c} \alpha_{h}=Q_{10}\cdot 0.07\cdot \text{Exp}\left(-\frac{V+65.0}{20.0}\right),\\ \beta_{h}=\frac{Q_{10}}{\left[1+\text{Exp}\left(-V+35.0/10.0\right)\right]}. \end{array}$$

(2) Nav1.5 sodium current:

(A.6) $$\begin{array}{c} I_{\text{Nav}1.5}=G_{\text{Nav}1.5}.m^{3}.h.\left(V-E_{\text{Na}}\right), \end{array}$$

  where *G*_Nav1.5_ is the maximum conductance. *m* and *h* are activation and inactivation variables. These can be expressed and solved from the ordinary differential equations showed in (A.6) and (A.8). *α*_*m*_, *β*_*m*_, *α*_*h*_, and *β*_*h*_ for Nav1.5 channel are denoted as

(A.7) $$\begin{array}{c} \alpha_{m}=\frac{Q_{10}\cdot 0.549\cdot \left(V+40.0\right)}{\left[1-\text{Exp}\left(-V+40.0/13.941\right)\right]},\\ \beta_{m}=Q_{10}\cdot 21.96\cdot \text{Exp}\left(-\frac{V+65.0}{6.2065}\right),\\ \alpha_{h}=Q_{10}\cdot 25.04\cdot \text{Exp}\left(-\frac{V+65.0}{7.2331}\right),\\ \beta_{h}=\frac{Q_{10}\cdot 1.445}{\left[1+\text{Exp}\left(-V-27.4554/7.1045\right)\right]}. \end{array}$$

(3) Nav1.9 sodium current:

(A.8) $$\begin{array}{c} I_{\text{Nav}1.9}=G_{\text{Nav}1.9}.m.h.s.\left(V-E_{\text{Na}}\right), \end{array}$$

  where *G*_Nav1.9_ is the maximum conductance. *m*, *h*, and *s* are activation, fast inactivation, and slow inactivation variables. *m* and *h* can be expressed and solved from the ordinary differential equations showed in (A.6) and (A.8). The slow inactivation variable *s* can be expressed as

(A.9) $$\begin{array}{c} \frac{\text{d}s}{\text{d}t}=\frac{\left(s_{\infty }-s\right)}{\tau_{s}}, \end{array}$$

  where *τ*_*s*_=1/[*α*_*s*_(*V*)+*β*_*s*_(*V*)] and *s*_*∞*_=1.029/[1+Exp(*V*+68.65/12)]. The *α*_*m*_, *β*_*m*_, *α*_*h*_, *β*_*h*_, *α*_*s*_, and *β*_*s*_ for Nav1.9 channel are

(A.10) $$\begin{array}{c} \alpha_{m}=\frac{Q_{10}\cdot 0.47}{\left[1+\text{Exp}\left(-V+15.426/10.3\right)\right]},\\ \beta_{m}=Q_{10}\cdot \text{Exp}\left(-\frac{V+61.426}{5.7}\right),\\ \alpha_{h}=\frac{Q_{10}\cdot 0.091}{\left[1+\text{Exp}\left(V+97.5095/18.8\right)\right]},\\ \beta_{h}=\frac{Q_{10}\cdot 0.03}{\left[1+\text{Exp}\left(-V+21.5095/10.0\right)\right]},\\ \alpha_{s}=Q_{10}\cdot 0.001\cdot \text{Exp}\left(-\frac{V+112.65}{17.5}\right),\\ \beta_{s}=\frac{Q_{10}\cdot 0.002}{\left[1+\text{Exp}\left(-V+1.65/21.0\right)\right]}. \end{array}$$

(4) Delayed rectifier potassium current:

(A.11) $$\begin{array}{c} I_{\text{Kdr}}=G_{\text{Kdr}}.n^{4}.\left(V-E_{\text{K}}\right), \end{array}$$

  where *G*_Kdr_ is the maximum conductance of the channel and *E*_K_=−89 mV is the reversal potential for potassium current. *N* is the activation variable that can be expressed as

(A.12) $$\begin{array}{c} \frac{\text{d}n}{\text{d}t}=\frac{\left(n_{\infty }-n\right)}{\tau_{n}}, \end{array}$$

  where *τ*_*n*_=1/(*α*_*n*_+*β*_*n*_), *n*_*∞*_=*α*_*n*_/(*α*_*n*_+*β*_*n*_), and

(A.13) $$\begin{array}{c} \alpha_{n}=\frac{Q_{10}\cdot 0.002199\cdot \left(V+12.902\right)}{\left[1-\text{Exp}\left(-V+12.902/7.5341\right)\right]},\\ \beta_{n}=Q_{10}\cdot 0.0087407\cdot \text{Exp}\left(-\frac{V+53.108}{7.4505}\right). \end{array}$$

(5) **I**_**K****M**_-Kv7 current:

(A.14) $$\begin{array}{c} I_{\text{KM}}=G_{\text{KM}}.n.\left(V-E_{K}\right), \end{array}$$

  where *G*_KM_ is the maximum conductance of the channel and *n* is the activation variable that can be expressed as

(A.15) $$\begin{array}{c} \frac{\text{d}n}{\text{d}t}=\frac{\left(n_{\infty }-n\right)}{\tau_{n}}, \end{array}$$

  where *τ*_*n*_=1/(*α*_*n*_+*β*_*n*_), *n*_*∞*_=1.0/[1+Exp(−*V*+30/6.0)], and

(A.16) $$\begin{array}{c} \alpha_{n}=Q_{10}\cdot 0.00395\cdot \text{Exp}\left(-\frac{V+30.0}{40.0}\right),\\ \beta_{n}=Q_{10}\cdot 0.00395\cdot \text{Exp}\left(-\frac{V+30.0}{20.0}\right). \end{array}$$

(6) N-type calcium current:

(A.17) $$\begin{array}{c} I_{\text{Ca}-N}=G_{\text{Ca}-N}.m^{2}.h.\left(V-E_{\text{Ca}}\right), \end{array}$$

  where *G*_Ca−*N*_ is the maximum conductance and *E*_Ca_=+132mV is the reversal potential for calcium current. *m* and *h* are activation and inactivation variables and can be expressed and solved from the ordinary differential equations shown in (A.6) and (A.8). *α*_*m*_, *β*_*m*_, *α*_*h*_, and *β*_*h*_ for N-type calcium channel are

(A.18) $$\begin{array}{c} \alpha_{m}=\frac{Q_{10}\cdot 0.1\cdot \left(V+20.0\right)}{\left[1-\text{Exp}\left(-V-20.0/10.0\right)\right]},\\ \beta_{m}=Q_{10}\cdot 0.4\cdot \text{Exp}\left(-\frac{V+25.0}{18.0}\right),\\ \alpha_{h}=Q_{10}\cdot 0.01\cdot \text{Exp}\left(-\frac{V+50.0}{10.0}\right),\\ \beta_{h}=\frac{Q_{10}\cdot 0.1}{\left[1+\text{Exp}\left(-V+17.0/17.0\right)\right]}. \end{array}$$

(7) Nonspecific current:

(A.19) $$\begin{array}{c} I_{h}=G_{h}.n.\left(V-E_{h}\right), \end{array}$$

  where *G*_*h*_ is the maximum conductance of the channel and *E*_*h*_=−28mV is the reversal potential for nonspecific current. *N* is the activation variable that can be expressed as

(A.20) $$\begin{array}{c} \frac{\text{d}n}{\text{d}t}=\frac{\left(n_{\infty }-n\right)}{\tau_{n}}, \end{array}$$

  where *τ*_*n*_=*Q*_10_ · 593 · Exp(0.4 · (*V*+73.0/9.0))/1+Exp(*v*+73.0/9.0), *n*_*∞*_=1.0/[1+Exp(*V*+78/11.0)],
(8) Passive leak current:

(A.21) $$\begin{array}{c} I_{\text{leak}}=G_{L}.\left(V-E_{L}\right), \end{array}$$

  where *G*_*L*_ is the maximum conductance of the channel and *E*_*L*_=−75mV is the reversal potential for nonspecific current.
(9) Mechanosensitive ionic current:
  The mechanosensitive ionic current can be denoted as [20]

(A.22) $$\begin{array}{c} I_{m}=p_{0}\cdot g_{m}.\left(V-E_{m}\right), \end{array}$$

  where *p*_0_=1.0/[1+Exp(*ε*_1/2_+*ε*/*S*_1/2_)] is the fraction of the channel that opens for a given strain and *ε*, *S*_1/2_, and *ε*_1/2_ are the strain of the neuron, steepness of the transition, and the strain associated with 50% of the channels in the open state. *g*_*m*_ and *E*_*m*_ are the conductance of the channel and the reversal potential for the mechanosensitive channel current. In this study, as there was a constant longitudinal stretch acting on the neuron during the mechanical stimulus, *p*_*0*_ can be simplified as a constant. Hence, equation (A.21) can be denoted as

(A.23) $$\begin{array}{c} I_{m}=G_{m}.\left(V-E_{m}\right), \end{array}$$

  where *G*_*m*_ is the maximum conductance of the channel.

## Abbreviations

IPANS: Intrinsic primary afferent neurons
AHP: After-hyperpolarization
sAHP: Slow after-hyperpolarization
mAHP: Medium after-hyperpolarization
MP: Membrane potential
RA: Rapidly accommodating
SA: Slowly accommodating.
